# Effectiveness and Safety of Herbal Decoctions for Functional Dyspepsia in Korean Medicine Clinics: A Multicenter Prospective Cohort Study Protocol

**DOI:** 10.3390/healthcare14152439

**Published:** 2026-08-06

**Authors:** Chaehyun Park, Na-Yeon Ha, Jinsung Kim

**Affiliations:** 1 Department of Clinical Korean Medicine, Graduate School, Kyung Hee University, Seoul 02447, Republic of Korea; chyun1602@naver.com; 2 Department of Digestive Diseases, College of Korean Medicine, Kyung Hee University, Seoul 02447, Republic of Korea; 3 Division of Digestive Diseases, Department of Korean Internal Medicine, Kyung Hee University Korean Medicine Hospital, Seoul 02447, Republic of Korea

**Keywords:** functional dyspepsia, herbal medicine, cohort study

## Abstract

**Objective**: Functional dyspepsia (FD) is frequently treated in Korean Medicine (KM) clinics in South Korea. With the recent inclusion of herbal decoctions (HDs) for FD in the national pilot program for health insurance coverage, the generation of real-world evidence has become an important policy priority. In this study, we aim to evaluate the real-world clinical outcomes and safety of individualized HD-centered KM care in patients with FD in a primary care setting. **Methods**: This multicenter, prospective, single-arm observational cohort study will enroll 150 participants who meet the Rome IV criteria for FD across 23 KM clinics nationwide. Participants will receive individualized HDs, prescribed at the discretion of the attending physicians, as the primary treatment for 4 weeks. Concomitant KM treatments (e.g., acupuncture, moxibustion, and cupping) are permitted as clinically needed to preserve the pragmatic nature of routine care. The primary outcome is the change in the total symptom score on the Nepean Dyspepsia Index, Korean version (NDI-K), at week 4. Secondary outcomes include the Numeric Rating Scale for overall severity, overall treatment effect, and safety monitoring. Statistical analyses will be performed using mixed models for repeated measures to account for repeated observations and missing data. **Discussion**: This study will evaluate the pragmatic outcomes of individualized HD-centered KM care in routine clinical practice, thereby supporting the external validity of its findings. The findings are expected to provide foundational real-world data for the national HD pilot program, and these data may serve as a reference for healthcare policies and clinical decision-making. Clinical Trial Registration: Clinical Research Information Service (registration number KCT0010254); registered on 11 February 2025.

## 1. Introduction

Functional dyspepsia (FD) is a chronic gastrointestinal disorder characterized by symptoms such as postprandial fullness, early satiation, epigastric pain, or epigastric burning. According to the Rome IV criteria, FD is diagnosed when these symptoms occur in the absence of any identifiable structural or organic disease that could explain them, provided that the symptoms begin at least 6 months prior to diagnosis and have been present during the preceding 3 months [1]. A recent large-scale systematic review and meta-analysis estimated the global prevalence of FD to be approximately 7.2% based on the Rome IV criteria [2]. In South Korea, dyspepsia ranked seventh among outpatient conditions in Korean Medicine (KM) clinics according to the 2024 Health Insurance Statistics Yearbook and has consistently remained within the top 10 over the past decade [3]. This sustained high ranking reflects the substantial and ongoing demand for KM treatment and highlights its contribution to the overall healthcare burden in South Korea.

The underlying mechanisms of FD are complex and heterogeneous. The key physiological drivers include gastric dysmotility (e.g., delayed emptying and compromised fundic accommodation), heightened visceral sensitivity, alterations in duodenal mucosal integrity, disturbances in the gut microbiome, and various psychological factors [4]. Given the multifactorial nature of FD, conventional therapies such as proton pump inhibitors (PPIs) and prokinetics often yield suboptimal outcomes, as they typically target only a single mechanism rather than the full spectrum of the disorder [5]. Moreover, the extended administration of pharmacological agents such as PPIs and prokinetics is frequently restricted by concerns about adverse effects, including cardiovascular and metabolic risks [6,7]. Consequently, many patients seek alternative treatment options, resulting in an increased demand for KM treatment.

In KM, FD is managed through individualized interventions, with herbal decoctions (HDs) serving as the core modality. Although various herbal medicines (HMs) have demonstrated clinical efficacy against FD in randomized controlled trials (RCTs) [8,9], these explanatory studies often have inherent limitations in their clinical applicability. Most existing RCTs have been conducted in highly controlled hospital-based settings with rigid inclusion criteria that do not fully account for the complexity of primary care. Specifically, the standardized protocols of traditional RCTs often overlook the individualized nature of KM, in which treatments are dynamically adjusted based on pattern identification (*Bian-Zheng*) and patient-centered factors [10]. Alternative designs such as pragmatic trials or cluster-randomized trials can provide comparative real-world evidence; however, they may still have limitations in fully reflecting the flexible, individualized nature of primary KM practice.

Recently, the South Korean government launched the second phase of a national pilot program to expand health insurance coverage, with FD newly included as a key target condition [11]. This policy shift underscores the need for high-quality real-world data on the utilization, clinical outcomes, and safety of HDs in routine practice. Given the individualized and multimodal nature of primary KM care, a multicenter prospective observational cohort study is well suited to capturing routine clinical outcomes. Therefore, this study aims to evaluate the real-world clinical outcomes and safety of individualized HD-centered KM care in patients with FD across 23 primary KM clinics. Although uncontrolled observational studies have inherent limitations—such as confounding, selection bias, and expectation bias—this study will generate valuable pragmatic evidence to support the ongoing national HD pilot program and offer practical insights for future health policy and clinical practice.

## 2. Methods

### 2.1. Study Registration

The study is registered with the Clinical Research Information Service under the registration number KCT0010254. URL (accessed on 7 July 2026): https://cris.nih.go.kr/cris/search/detailSearch.do?seq=29160&status=5&seq_group=29160&search_page=M.

### 2.2. Study Design

This is a multicenter, prospective observational cohort study designed to collect real-world data from routine clinical practice without randomization or blinding. To better reflect routine clinical practice, participants will receive assessments and treatments as part of routine clinical care based on the physician’s judgment. The study will be conducted at 23 KM clinics in different regions of South Korea (Table S1), with Kyung Hee University Korean Medicine Hospital serving as the coordinating center. Participants will undergo individualized KM treatments as determined by their physician, followed by clinical assessments and patient-reported outcome surveys at baseline (week 0), visit 2 (week 2), and visit 3 (week 4). This protocol was developed and reported in accordance with the Standard Protocol Items: Recommendations for Interventional Trials (SPIRIT) guidelines (Table S2).

### 2.3. Study Participants

#### 2.3.1. Recruitment

Participants will be recruited voluntarily from 23 KM clinics during routine clinical practice. No predefined recruitment quotas will be assigned to the participating centers. Physicians will provide patients who meet the eligibility criteria (Table 1) with a comprehensive information document detailing the scope of the study. Written informed consent will be obtained before any research-related activities commence.

#### 2.3.2. Withdrawal

Participant participation may be terminated if any one of the following criteria are met:

1. Noncompliance with eligibility criteria discovered after enrollment.
2. Withdrawal of consent by the participant or legal guardian.
3. Investigator’s clinical judgment that continued participation is inappropriate.

### 2.4. Interventions

Because this is an observational study, no experimental intervention is assigned or controlled by the researchers. During the 4-week observation period, participants will receive individualized KM treatments, defined as “HD-centered care,” in which tailored HDs serve as the primary therapeutic modality, supplemented by routine individualized KM care as dictated by the attending physician.

Although HDs are flexibly individualized based on pattern identification, representative formulas frequently prescribed in primary care for FD include Banha-sasim-tang, Yukgunja-tang, and Hyangsa-pyeongwi-san [8,9,12]. These traditional formulas are well documented for improving gastrointestinal motility, modulating visceral hypersensitivity, and protecting the mucosal barrier through key bioactive compounds, and they have a well-established safety profile in clinical practice.

In principle, HDs are prescribed in standard dispensing units (typically comprising 20 therapeutic doses yielding 30 packs, designated for administration three times daily), though these may be adjusted according to individual clinical conditions and practice environments. Detailed prescriptions and dosage information—including repeated prescriptions—will be recorded, and patient adherence to HDs will be monitored based on patient-reported remaining pack counts during follow-up assessments.

Other KM treatments administered as part of routine care, such as herbal extract granules, acupuncture, electroacupuncture, moxibustion, cupping therapy, and pharmacopuncture, may also be provided at the treating physician’s discretion. Detailed information on all KM interventions and concomitant medications, including prescription duration, administration frequency, and concomitant treatments, will be thoroughly documented in the electronic case report form (eCRF) to capture treatment exposure and heterogeneity.

### 2.5. Outcome Measures

Clinical assessments are scheduled at baseline (week 0), week 2, and week 4. For participants who are unable to visit the clinic in person at week 2 or 4, assessments may be conducted via telephone interviews to minimize missing data and ensure continuity. Missing outcome data will not be imputed and will be managed within the mixed models for repeated measures (MMRMs).

#### 2.5.1. Primary Outcome Measure

The primary outcome is the change in the total symptom score of the Korean version of the Nepean Dyspepsia Index (NDI-K) from baseline to week 4. The NDI-K is a validated tool for quantifying the severity of dyspeptic symptoms and their impact on patient well-being. This study will use a 15-item symptom subscale that captures the frequency and intensity of discomfort; higher cumulative values represent a greater symptom burden [13]. As the original NDI comprises distinct symptom and quality-of-life subscales, this study specifically uses the 15-item Nepean Dyspepsia Symptom Index (NDSI) as the primary outcome measure to precisely evaluate core physical discomforts associated with FD, independent of general quality-of-life domains. In accordance with established clinical trial standards employing the NDSI [14], therapeutic efficacy will be evaluated primarily through continuous score reductions in this symptom subscale.

#### 2.5.2. Secondary Outcome Measures

- Individual NDI-K Scores: Changes in the scores of each of the 15 symptom items.
- Numeric Rating Scale (NRS): An 11-point scale (0–10) assessing overall dyspepsia severity.
- Overall Treatment Effect (OTE): A 7-point Likert scale assessing global improvement. Participants classified as “much improved” or “very much improved” on the OTE scale will be defined as treatment responders, and the proportion of responders at week 4 will be analyzed as a secondary outcome [15].

### 2.6. Study Procedure

The study procedure is summarized in Table 2. The total study duration for each participation is 4 weeks.

#### 2.6.1. Screening and Consent

Physicians will explain the research objectives in detail, including the potential risks and therapeutic expectations. Written informed consent will be obtained before collecting any study-specific data. Baseline data encompass comprehensive medical histories, including preexisting conditions and current medication profiles. To satisfy the endoscopy requirement, documentation of an upper gastrointestinal endoscopy performed after symptom onset must be verified. Candidates without a documented post-symptom-onset endoscopy are ineligible for enrollment.

#### 2.6.2. Baseline Visit (Week 0)

After providing written informed consent, participants will undergo a baseline assessment. During this visit, researchers confirm eligibility, assign a unique identification code to ensure confidentiality, and collect baseline data via the eCRF.

The collected data include demographic characteristics (sex, date of birth, and age), anthropometric measurements (height, weight, and body mass index [BMI]), and medical history, including relevant comorbidities and concomitant medications (drug name, dosage, route of administration, indication, and duration of use).

For FD-related history, information regarding symptom onset, prior diagnostic evaluations (including upper gastrointestinal endoscopy findings), previous treatments and responses, and baseline disease characteristics will be documented in detail. Baseline outcome measures include the NRS for dyspepsia severity and the NDI-K.

#### 2.6.3. Treatment and Follow-Up Visits

Participants will return for follow-up evaluations at weeks 2 and 4. These visits involve updating the NDI-K and NRS scores, reviewing changes in herbal prescriptions, and documenting concomitant therapies or adverse events to track the longitudinal impact of treatment. Patient adherence to HDs will be systematically assessed at each follow-up visit based on patient-reported counts of remaining pouches. Unscheduled visits, defined as visits occurring when participants present to the clinic outside of scheduled visit time points, will also be documented to capture all relevant clinical data.

### 2.7. Safety Assessment

Adverse events (AEs) will be monitored at each visit. All AEs will be recorded in detail, including the onset, duration, severity, outcome, and management. AEs will be categorized using version 5.0 of the Common Terminology Criteria for Adverse Events (CTCAE). The causal relationship between AEs and HDs will be assessed independently by the investigators based on their clinical judgment. If necessary, external expert consultation may be sought to confirm the causality assessment. All AEs will be managed with appropriate clinical care until resolution. Any serious adverse event (SAE) must be reported to the Principal Investigator and IRB within 24 h of awareness. As this study reflects routine primary care practice, protocol-mandated laboratory safety tests will not be routinely collected.

### 2.8. Data Collection and Management

Data will be collected using designated research worksheets and patient-reported outcome questionnaires. All clinical information and patient responses recorded on paper worksheets will be entered into a centralized and secure eCRF system by the investigators at each site. The eCRF platform features automated validation rules to prevent data entry errors.

A Data Management Officer at the coordinating center conducts periodic audits. If discrepancies arise, the site Principal Investigator (Clinic Director) at the respective site will be notified to verify the source documents and rectify the electronic records.

All physical worksheets and source documents containing personal information will be pseudonymized using a unique participant identification code and will be transferred to the coordinating center in sealed, secure envelopes. Access to these materials will be restricted to authorized research personnel. Upon receipt, all documents will be stored in locked cabinets in restricted-access rooms to ensure confidentiality. The eCRF data will be stored on a password-protected, encrypted server with restricted access rights. All study materials will be archived for at least five years in accordance with national bioethics and data retention requirements.

### 2.9. Sample Size

This exploratory multicenter observational cohort study will be conducted under routine clinical conditions. Because of the lack of prior data on NDI-K changes in this specific clinical context, a formal power calculation was not performed. Instead, the target sample size was determined based on the design of previous clinical trials for HMs in FD [16] and recruitment feasibility across 23 participating KM clinics.

The dropout rate was conservatively set at 25%. Although previous multicenter clinical trials on FD in KM typically assumed a 20% attrition rate [17], a higher threshold is adopted to reflect the pragmatic nature of this study. Assuming a 25% dropout rate, recruiting 150 participants will yield approximately 112 evaluable participants, ensuring our goal of securing at least 100 fully evaluable cases for the final analysis. Furthermore, based on prior literature in which the standard deviation (SD) of NDI-K score changes was approximately 10–12 points [16], a sample of 112 evaluable participants provides high statistical precision. The expected margin of error for the mean primary outcome change is approximately 1.85–2.22 points at the 95% confidence level, providing reliable parameter estimates for this exploratory study.

### 2.10. Statistical Analysis

Statistical significance will be set at *p* < 0.05. Baseline characteristics will be reported using mean ± SD for continuous data, while categorical data will be summarized as frequencies and percentages.

To assess clinical effectiveness, longitudinal changes in NDI-K and NRS scores across time points will be modeled using MMRM. Specifically, for the primary outcome (change in NDI-K score at week 4), the MMRM will fit categorical visit (weeks 2 and 4), baseline NDI-K score as a continuous covariate, and a visit-by-baseline interaction as fixed effects. The inclusion of week 2 data allows utilization of all longitudinal observations to stabilize covariance estimation and provide unbiased estimates of week 4 changes. An unstructured variance-covariance matrix (or a heterogeneous first-order autoregressive structure [ARH(1)] if the model does not converge) will be applied.

To account for clinic-level clustering and intra-center correlations across the 23 participating clinics, participating sites will be included as a random effect in the mixed models. Furthermore, to accommodate potential confounding factors and treatment heterogeneity in routine clinical practice—such as baseline clinical characteristics, symptom severity, and concomitant KM interventions—multivariable regression models incorporating these key variables as covariates will be performed. Additionally, exploratory subgroup analyses based on these clinical factors may be conducted to evaluate their potential modifying effects. This approach effectively addresses within-subject correlations and handles missing data under the missing-at-random (MAR) assumption; consequently, no explicit data imputation is performed in the primary model. To test the robustness of the primary outcomes against potential departures from the MAR assumption (e.g., missing not at random), sensitivity analyses will be conducted using multiple imputation (MI) under MAR and pattern mixture models (PMMs) with delta-adjustment. Sensitivity analyses will also be conducted using the per-protocol set (PPS) to verify that the missing data pattern does not substantially alter the main findings. Additionally, paired *t*-tests will be performed as a supplementary analysis to evaluate overall within-subject pre- and post-treatment changes. Furthermore, potential assessment mode differences (in-person vs. telephone follow-up) will be evaluated by including assessment mode as a covariate or subgroup factor in secondary analytic models.

Safety monitoring data will be descriptively summarized, focusing on the frequency, intensity, and causal relationships of any recorded AEs. Exploratory subgroup analyses will also be performed based on whether participants received concomitant KM interventions. Where applicable, differences in the AE incidence rates between these subgroups will be evaluated using the chi-square test or Fisher’s exact test.

The full analysis set (FAS) will serve as the primary population for effectiveness outcomes, whereas the PPS and safety analysis set (SAS) will be used for per-protocol and safety evaluations, respectively. The specific criteria for these sets of analyses are outlined below.

- FAS: This dataset will comprise all enrolled participants who satisfied the eligibility criteria, received HD on at least one occasion, and provided at least one post-baseline effectiveness measurement.
- PPS: This subset will consist of FAS participants who successfully completed the entire follow-up period without committing any major protocol violations or noncompliance defined a priori.
- SAS: This population will include all registered individuals who received at least one HD dose and will undergo subsequent safety monitoring.

### 2.11. Status and Timeline of the Study

Participant recruitment commenced on 25 February 2025 and is currently ongoing. Recruitment is expected to be completed by 31 December 2028, and final data collection is anticipated by January 2029.

## 3. Discussion

Although FD is prevalent in KM clinics, prospective, real-world evidence remains limited. Although several multicenter studies have investigated FD, most have been explanatory RCTs focusing on standardized herbal formulas in controlled hospital settings [16,18]. To the best of our knowledge, this prospective observational cohort study is designed to provide valuable real-world evidence on the clinical outcomes and safety of individualized HDs administered in routine primary care.

Unlike traditional RCTs, this study adopts a pragmatic observational design that reflects routine clinical practice, in which treatment strategies are tailored to individual patients and often combined with other KM modalities. By prospectively collecting data from 23 primary KM clinics, this study is expected to provide clinically meaningful insights into current prescription patterns, treatment responses, and safety outcomes in routine clinical settings.

This study has several important policy implications. In South Korea, the national pilot program for health insurance coverage of HDs was recently expanded to include FD as a target condition [11]. Despite the widespread clinical use of HDs in the treatment of gastrointestinal disorders, prospective real-world evidence remains insufficient. Therefore, this study will generate valuable, hypothesis-generating data to support the ongoing national pilot program and inform future healthcare policies and clinical decision-making.

This study has several inherent methodological limitations. First, because this is an observational study without a randomized controlled group, a causal relationship between HD treatment and clinical outcomes cannot be established. Second, the inherent heterogeneity of individualized prescriptions and concomitant KM interventions may complicate the interpretation of the treatment effects. To address potential confounding and heterogeneity, multivariable regression analyses and exploratory subgroup analyses based on concomitant KM interventions will be performed. Third, the 4-week observation period may be insufficient to evaluate the long-term effectiveness and recurrence patterns of FD. Furthermore, loss to follow-up and missing data may occur due to pragmatic primary care factors, such as early symptom resolution, scheduling conflicts, or noncompliance. Finally, because the study relies primarily on patient-reported outcomes in an open-label setting, reporting and expectation biases cannot be excluded.

Despite these limitations, this study provides a practical, standardized framework for generating pragmatic real-world evidence on HD-based care for FD in primary KM settings. This study will serve as foundational, hypothesis-generating evidence to support future pragmatic clinical research and evidence-based healthcare policies integrating KM into public health systems.

## Figures and Tables

**Table 1:** Eligibility criteria.

**Inclusion Criteria**
Adults aged 19–74 years. Patients diagnosed with FD according to the Rome IV criteria, presenting with at least one symptom (postprandial fullness, early satiation, epigastric pain, or epigastric burning) that started at least 6 months prior and persisted for the last 3 months, without structural findings explaining the symptoms, as confirmed by upper gastrointestinal endoscopy performed after symptom onset. Patients who voluntarily provide written informed consent using a form approved by the Institutional Review Board (IRB).
**Exclusion Criteria**
Organic diseases (e.g., peptic ulcer, gastroesophageal malignancy, or pancreaticobiliary disease) identified by endoscopy or other diagnostic tests performed after symptom onset. History of major gastrointestinal surgery (excluding appendectomy > 6 months prior). Presence of alarm symptoms (e.g., unexplained weight loss, hematochezia, dysphagia, or persistent vomiting). Severe underlying pathologies affecting the cardiac, pulmonary, hepatic, or renal systems. Significant neuropsychiatric history (e.g., major depressive disorder or schizophrenia). Pregnant or lactating women. History of drug or alcohol abuse. Participation in other clinical trials that may interfere with this study.

**Table 2:** Participant timeline: Schedule of enrollment and assessments.

	STUDY PERIOD				
TIMEPOINT (Week ± Day)	SCREENING (~1 week)	Visit 1 (0)	Visit 2 (2 weeks ± 7 days)	Visit 3 (4 weeks ± 7 days)	Unscheduled Visit
**ENROLLMENT:**					
Eligibility screen	X				
Informed consent	X				
Demographic/socioeconomic survey	X				
Medical history and treatment history	X				
Physical measurements	X				
**ASSESSMENTS:**					
Overall treatment effect			X	X	
Numeric Rating Scale		X	X	X	
Nepean Dyspepsia Index-Korean		X	X	X	
Korean medicine treatment record		X	X	X	X
Concomitant medication		X	X	X	X
Adverse events		X	X	X	X

## Data Availability

As this paper is a protocol for a clinical study, no data have been collected thus far; therefore, there are no data to share.

## Supplementary Materials

The following supporting information can be downloaded at: https://www.mdpi.com/article/10.3390/healthcare14152439/s1, Table S1: Participating Korean medicine clinics. Table S2: SPIRIT 2025 editable checklist. Reference [19] is cited in the Supplementary Materials.

## Abbreviations

The following abbreviations are used in this manuscript:

FD	Functional dyspepsia
HD	Herbal decoction
HM	Herbal medicine
KM	Korean medicine
MMRMs	Mixed models for repeated measures
NDI-K	Nepean Dyspepsia Index, Korean version
NRS	Numeric Rating Scale
OTE	Overall treatment effect
RCT	Randomized controlled trial
