# Supplementary material for: Effectiveness and Safety of Herbal Decoctions for Functional Dyspepsia in Korean Medicine Clinics: A Multicenter Prospective Cohort Study Protocol
Source: Healthcare (Basel). 2026 Aug 6;14(15):2439. doi: 10.3390/healthcare14152439 (PMC13466125; doi:10.3390/healthcare14152439)
Supplement: Supplementary file 1 [file healthcare-14-02439-s001.zip › healthcare-4453251-supplementary/Table S1. Participating KM clinics.pdf]

Table S1. Participating Korean medicine clinics.

Clinic	Region
Plus Kyunghee Korean Medicine Clinic	Uiwang
Tokbarun Kyunghee Korean Medicine Clinic	Seoul
Wegang Korean Medicine Clinic Guri-Namyangju Branch	Guri
Wirye Chacha Korean Medicine Clinic	Seoul
Yeonwoodang Korean Medicine Clinic	Seoul
Kyungheeroun Korean Medicine Clinic	Seoul
Hyoin Korean Medicine Clinic	Seoul
Kyunghee 365 Korean Medicine Clinic	Gimhae
Gil Union Korean Medicine Clinic	Daegu
Kyunghee Sweet & Warm Korean Medicine Clinic	Namyangju
Kyunghee Mirae Korean Medicine Clinic Boramae Branch	Seoul
Wegang Korean Medicine Clinic Bundang Main Branch	Seongnam
Suseong Misodam Korean Medicine Clinic	Daegu
Jangwi 365 Kyunghee Korean Medicine Clinic	Seoul
Yeokjeon Cham Korean Medicine Clinic	Jeonju
Jungdong Korean Medicine Clinic	Bucheon
Chewon Korean Medicine Clinic	Seoul
Mullae Majubom Korean Medicine Clinic	Seoul
Buyeo Jangsu Korean Medicine Clinic	Buyeo
Jami Korean Medicine Clinic	Ansan
Haemalgeun Korean Medicine Clinic	Cheonan
Jangssi Samdae Korean Medicine Clinic	Osan
Sumac Korean Medicine Clinic	Anyang
